# Validation for measurements of skeletal muscle areas using low-dose chest computed tomography

**DOI:** 10.1038/s41598-021-04492-1

**Published:** 2022-01-10

**Authors:** Woo Hyeon Lim, Chang Min Park

**Affiliations:** 1 Department of Radiology, Namwon Medical Center, Namwon-si, Jeollabuk-do Korea; 2 Department of Radiology, Seoul National University College of Medicine, 101 Daehak-ro, Chongno-gu, Seoul, 03080 Korea; 3 Institute of Radiation Medicine, Seoul National University Medical Research Center, Seoul, Korea; 4 Cancer Research Institute, Seoul National University College of Medicine, Seoul, Korea; 5 Institute of Medical and Biological Engineering, Medical Research Center, Seoul National University, Seoul, Korea

**Keywords:** Population screening, Medical imaging

## Abstract

Various methods were suggested to measure skeletal muscle areas (SMAs) using chest low-dose computed tomography (chest LDCT) as a substitute for SMA at 3rd lumbar vertebra level (L3-SMA). In this study, four SMAs (L1-SMA, T12-erector spinae muscle areas, chest wall muscle area at carina level, pectoralis muscle area at aortic arch level) were segmented semi-automatically in 780 individuals taking concurrent chest and abdomen LDCT for healthcare screening. Four SMAs were compared to L3-SMA and annual changes were calculated from individuals with multiple examinations (n = 101). Skeletal muscle index (SMI; SMA/height^2^) cut-off for sarcopenia was determined by lower 5th percentile of young individuals (age ≤ 40 years). L1-SMA showed the greatest correlation to L3-SMA (men, R^2^ = 0.7920; women, R^2^ = 0.7396), and the smallest annual changes (0.3300 ± 4.7365%) among four SMAs. L1-SMI cut-offs for determining sarcopenia were 39.2cm^2^/m^2^ in men, and 27.5cm^2^/m^2^ in women. Forty-six men (9.5%) and ten women (3.4%) were found to have sarcopenia using L1-SMI cut-offs. In conclusion, L1-SMA could be a reasonable substitute for L3-SMA in chest LDCT. Suggested L1-SMI cut-offs for sarcopenia were 39.2cm^2^/m^2^ for men and 27.5cm^2^/m^2^ for women in Asian.

## Introduction

Sarcopenia, characterized by decreased in muscle strength, quantity, and quality^1^, is garnering attention in medical fields including oncology^2–4^, pulmonology^5^, and cardiology^6^. Older adults are vulnerable to sarcopenia as well^7^, and they have often undergone chest low-dose computed tomography (LDCT) for lung cancer screening^8^, and diagnosis and monitoring for other various pulmonary pathologies including lung cancer, interstitial lung diseases (ILD)^9^, and chronic obstructive pulmonary disease (COPD)^10^. Unfortunately, however, typical chest LDCT does not cover 3rd lumbar vertebra (L3) level, which is considered as a reference standard for sarcopenia diagnosis for body imaging-based study^2–6^.

Thus, investigators tried to utilize various anatomic landmarks as substitutes for skeletal muscle area (SMA) at L3 level (L3-SMA) in chest CT: (1) SMA at 1st lumbar vertebral body (L1) level (L1-SMA)^11–13^, (2) erector spinae muscle area (ESMA) at 12th thoracic vertebral body (T12) level (T12-ESMA)^14–16^, (3) chest wall muscle area (CWMA) at carina (C-CWMA)^17^ or 4th thoracic vertebral body (T4) level^15^ where carina is usually located, and (4) pectoralis muscle area (PMA) just above aortic arch level (AA-PMA)^11,13,14,18^.

However, comprehensive comparison between L3-SMA and multiple SMAs previously suggested as substitutes for L3-SMA using chest CT does not exist so far. Through this comparison, researchers could obtain more objective information on each SMA and standardize the utilization of chest CT in sarcopenia study. Furthermore, additional information such as race-specific cut-offs for each SMA in chest LDCT or whether the skeletal muscles decreased evenly according to their location could be obtained in general population-based study^19^.

Through this study, it is firstly aimed to suggest the optimal substitution upon chest LDCT examinations for L3-SMA and present Asian specific cut-offs for sarcopenia diagnosis. Second, factors related to uneven decrease of skeletal muscles, if so, were analyzed.

## Results

Table 1 summarizes demographics, anthropometric information and clinical information of 780 individuals included in this study.

**Table 1:** Information on 780 individuals taking healthcare screening examination.

	Men (n = 482)	Women (n = 298)
Age (years)	50.9 ± 10.5	51.4 ± 10.8
	(median, 51.0)	(median, 52.0)
	(range, 23.0–77.0)	(range, 21.0–79.0)
Body weight (kg)	72.2 [65.3, 79.9]	57.9 [52.5, 63.8]
Height (cm)	169.7 ± 6.1	157.3 ± 6.1
Body-mass index (kg/m^2^)	25.2 [23.2, 27.3]	23.3 [21.7, 25.7]
Waist circumference (cm)*	84.0 [79.0, 89.0]	75.0 [69.8, 80.3]
Abdominal obesity*	104 (21.7%)	45 (15.2%)
**Smoking**		
Never smoker	265 (55.0%)	294 (98.7%)
Ex-smoker	19 (3.9%)	1 (0.3%)
Current smoker	198 (41.1%)	3 (1.0%)
**Underlying disease**		
Hypertension	113 (23.4%)	48 (16.1%)
Diabetes mellitus	63 (13.1%)	22 (7.4%)
Dyslipidemia	87 (18.0%)	47 (15.8%)

Numerical variables with normal distribution were provided with mean ± standard deviation, while those not showing normal distribution were provided with median [interquartile range].

Abdominal obesity was defined as a waist circumference ≥ 90 cm in men, and ≥ 85 cm in women.

*These values were extracted from 776 individuals consisting of 479 men and 297 women.

### Comparison of chest LDCT-derived muscle areas with L3-SMA

**Figure 1:**
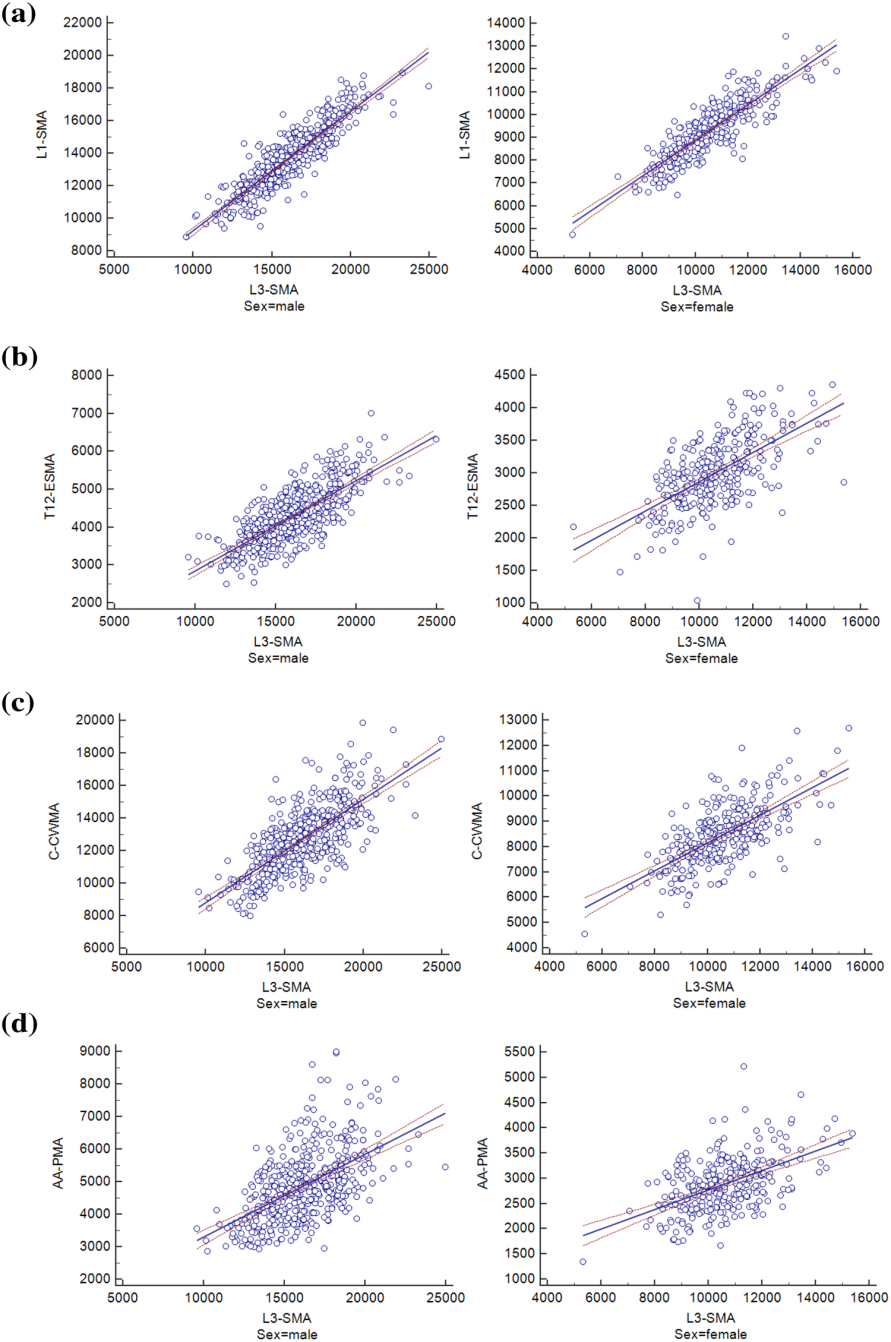
Correlation of skeletal muscle area (SMA) between L3-SMA and variable anatomic landmarks in chest CT: (**a**) L1-SMA (men, R^2^ = 0.7920; women, R^2^ = 0.7396), (**b**) T12-ESMA (men, R^2^ = 0.5557; women, R^2^ = 0.3834), (**c**) C-CWMA (men, R^2^ = 0.5306; women, R^2^ = 0.4427), and **d)** AA-PMA (men, R^2^ = 0.3052; women, R^2^ = 0.2672).

**Figure 2:**
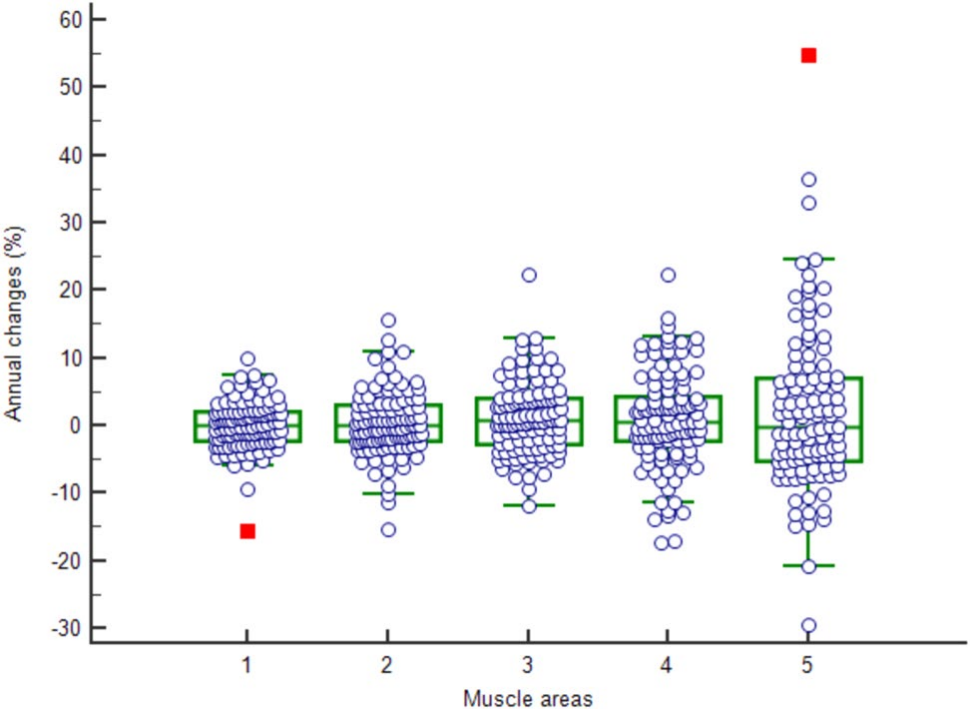
Box-and-Whisker plots regarding annual changes (%) of variable muscle areas ([1] skeletal muscle area at 3rd lumbar vertebra level, 0.0725 ± 3.5308%; [2] skeletal muscle area at 1st lumbar vertebra level, 0.3300 ± 4.7365%; [3] = erector spinae muscle area at 12th thoracic vertebra level, 1.1614 ± 5.3488%; [4] chest wall muscle area at carina level, 0.8622 ± 7.1192% [5]; pectoralis muscle area just above aortic arch level, 2.0202 ± 11.8910%).

Among four chest CT-derived muscle areas, L1-SMA (men, R^2^ = 0.7920; women, R^2^ = 0.7396) showed the greatest correlation to L3-SMA, while AA-PMA (men, R^2^ = 0.3052; women, R^2^ = 0.2672) exhibited the least correlation to L3-SMA (Fig. 1). L1-SMA showed the least annual changes and standard variation (0.3300 ± 4.7365%) among four areas (Fig. 2). The T12-ESMA and C-CWMA were influenced by reconstruction methods (*P* < 0.05).

### Chest LDCT-based definition of sarcopenia

Trends of all SMAs and SMIs according to age were described in Supplementary Fig. 1. The cut-off for L1-skeketal muscle index (L1-SMI) for sarcopenia was 39.2cm^2^/m^2^ for men, and 27.5cm^2^/m^2^ for women, respectively (Table 2). With this cut-off, 46 men (9.5%) and 10 women (3.4%) were found to have sarcopenia. Agreements to L3-SMI for defining sarcopenia were substantial for L1-SMI (κ = 0.622), moderate for C-CWMI (κ = 0.550) and AA-PMI (κ = 0.439), while T12-ESMI showed fair agreement (κ = 0.361) (Table 2).

**Table 2:** The cut-offs to define sarcopenia in Asian population.

	Men	Women	Sarcopenia (n)
L3-SMA (cm^2^) / SMI (cm^2^/m^2^)	142.1/46.3	85.6/32.8	55 (7.1%; M:W = 48:7)
L1-SMA (cm^2^) / SMI (cm^2^/m^2^)	116.2/39.2	73.7/27.5	56 (7.2%; M:W = 46:10)
T12-ESMA (cm^2^) / ESMI (cm^2^/m^2^)	34.3/11.6	22.9/8.4	49 (6.3%; M:W = 39:10)
C-CWMA (cm^2^) / CWMI (cm^2^/m^2^)	114.2/36.2	74.1/26.6	68 (8.7%; M:W = 53:15)
AA-PMA (cm^2^) / PMI (cm^2^/m^2^)	40.2/13.2	19.2/6.9	77 (9.9%; M:W = 72:5)

SMA = skeletal muscle area (L3 = at 3rd lumbar vertebra level, L1 = at 1st lumbar vertebra level), SMI = skeletal muscle index (L3 = at 3rd lumbar vertebra level, L1 = at 1st lumbar vertebra level), T12-ESMA = erector spinae muscle area at 12th thoracic vertebra level, T12-ESMI = erector spinae muscle index at 12th thoracic vertebra level, C-CWMA = chest wall muscle area at carina level, C-CWMI = chest wall muscle index at carina level, AA-PMA = pectoralis muscle area just above aortic arch level, AA-PMI = pectoralis muscle index just above aortic arch level, M = men, W = women.

### Risk factors for sarcopenia based on chest LDCT

In univariate logistic regression, sex, body weight, height, body-mass index (BMI), smoking status and factors related to central obesity (waist circumference [WC], visceral fat area [VFA], subcutaneous fat area [SFA], total fat area [TFA]) were found to be associated with sarcopenia based on L1-SMI (Table 3). In multivariate logistic regression, sarcopenia based on L1-SMI was significantly related with men (adjusted odds ratio [OR] = 25.856, 95th percentile confidence interval [95% CI] = 8.714 to 76.720, *P* < 0.001), lower BMI (OR = 0.336, 95% CI = 0.251 to 0.449, *P* < 0.001), and greater VFA (OR = 1.013, 95% CI = 1.001 to 1.025, *P* = 0.030). When VFA was replaced by SFA or TFA, men, lower BMI, and central obesity were persistently related with sarcopenia (*P* < 0.05).

**Table 3:** Univariate logistic regression analyses for evaluating factors related with sarcopenia defined by skeletal muscle index at 1st lumbar vertebra level (L1-SMI).

Variables	Sarcopenia ( +) (n = 56)	Sarcopenia (-) (n = 724)	Odds ratio (95th C.I)	*p*-value
Age (years)	52.5 ± 9.5	51.0 ± 10.6	1.014 (0.988, 1.040)	0.309
Sex (M:W)(n)	46 : 10	436 : 288	3.039 (1.509, 6.118)	0.002
Body weight (kg)	59.8 [54.0, 64.5]	67.6 [59.4, 76.4]	0.925 (0.900, 0.951)	< 0.001
Height (cm)	168.3 [163.9, 173.7]	165.3 [158.5, 171.3]	1.061 (1.026, 1.098)	< 0.001
BMI (kg/m^2^)	20.7 [19.6, 21.8]	24.8 [22.8, 27.1]	0.542 (0.468, 0.627)	< 0.001
**Smoking**				
Smoking status (n)				0.01
Current vs. Never	23:30	178 : 529	2.279 (1.290, 4.026)	0.005
Ex vs. Never	3 : 30	17 : 529	3.119 (0.864, 11.207)	0.083
PY (n = 696; 46:650)	7.9 ± 12.8	4.1 ± 10.2	1.027 (1.004, 1.050)	0.02
Hypertension (n)	7	154	0.529 (0.235, 1.191)	0.124
Diabetes mellitus (n)	6	79	0.980 (0.407, 2.358)	0.964
Dyslipidemia (n)	5	129	0.452 (0.177, 1.155)	0.097
Central obesity*				
WC (cm)	73.7 ± 6.9	81.3 ± 8.9	0.899 (0.867, 0.932)	< 0.001
VFA (cm^2^)	59.5 [37.0, 97.4]	105.7 [66.7, 143.8]	0.983 (0.977, 0.990)	< 0.001
SFA (cm^2^)	92.4 [61.5, 125.1]	150.6 [119.1, 192.3]	0.973 (0.965, 0.980)	< 0.001
TFA (cm^2^)	162.2 ± 67.3	266.0 ± 91.4	0.986 (0.982, 0.990)	< 0.001

Numerical variables with normal distribution were provided with mean ± standard deviation, while those not showing normal distribution were provided with median [interquartile range] except pack-year.

M = men, W = women, n = number, BMI = body-mass index, PY = Pack-Year, WC = waist circumference, FA = fat area (V:visceral, S:subcutaneous, T:total).

*Numbers of evaluated individuals (WC, n = 776, 54:722; VFA, n = 779, 55:724; SFA, n = 739, 55:684; TFA, n = 739, 55:684).

### Factors related to accelerated decrease of chest wall muscle areas

**Figure 3:**
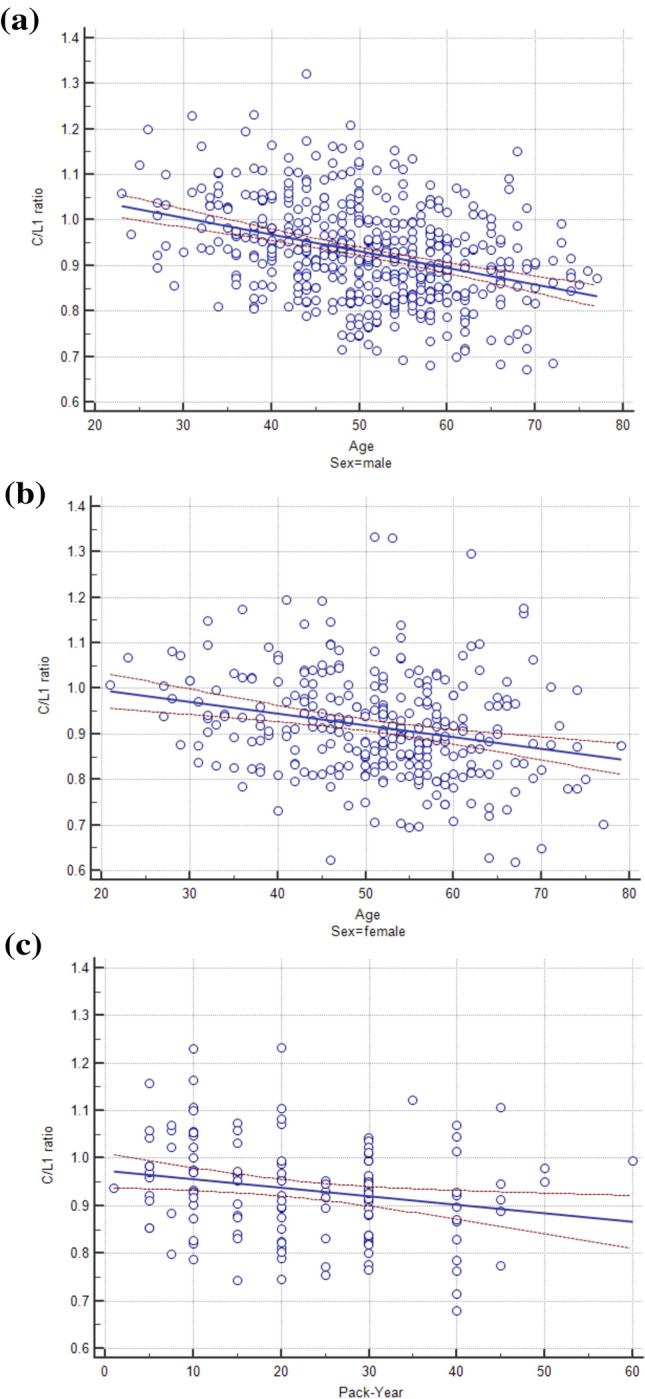
The C/L1 ratio (the ratio of chest wall muscle area at carina and skeletal muscle area at 1st lumbar vertebral level) according to age and smoking status: (**a**, **b**) The C/L1 ratio decreased with age (*P* < 0.001) in general population, and (**c**) pack-year (*P* = 0.012) in current smokers.

The lower C-CWMA/L1-SMA ratio (C/L1 ratio) was significantly associated with older age (*P* < 0.001) (Fig. 3), women (*P* = 0.007), and greater BMI (*P* < 0.001), while L3-SMI was not the factors related with C/L1 ratio. In addition, current smokers showed lower C/L1 ratio compared to never smokers after adjustment of age, sex, and BMI (*P* = 0.042), and this relationship was dose-dependent in current smokers with exact pack-year (PY) (n = 137, *P* = 0.012) (Fig. 3). Smoking status was not associated with L1-SMI after adjustment of age, sex, BMI, and VFA.

## Discussion

In this study, four representative anatomic landmarks were selected (L1-SMA, T12-ESMA, C-CWMA, and AA-PMA), because those were well validated in several studies^11–18^. The T12-SMA^20^ was not evaluated in this study, because T12-SMA which was smaller than L1-SMA, showed significant correlation with L1-SMA^21^. Besides, the utilization of intercostal muscles alone^22^ was not considered because their small areas could be vulnerable to measurement errors. Among four regions of interest, L1-SMA showed the greatest correlation to L3-SMA regardless of sex, while annual change was the smallest. In addition, in defining sarcopenia using region-specific cut-offs, L1-SMI provided the greatest agreement with L3-SMI. These findings suggested that L1-SMA could be a reasonable substitute for L3-SMA in chest CT-based study.

In this study, the importance of cut-offs to define sarcopenia in imaging-based study was also reaffirmed. One of the most commonly used cut-offs was those suggested by Prado et al. (men, 55.0cm^2^/m^2^; women, 39.0cm^2^/m^2^), which focused on prognostication in oncologic patients^2^. Although the prediction of prognosis in a specific disease is important, to understand age-dependent changes and diagnose sarcopenia in general population, the cut-offs of sarcopenia should be determined based on healthy general population^21^, which is similar to T-score in osteoporosis.

Kim et al. suggested L1-SMI cut-offs (men, 46.0cm^2^/m^2^; women, 29.0cm^2^/m^2^), which were calculated from linear regression between L3-SMI and L1-SMI^13^. Because Kim et al^13^ used predefined L3-SMI cut-offs from Prado et al^2^, these L1-SMI cut-offs were quite greater than ours (men, 39.2cm^2^/m^2^; women, 27.5cm^2^/m^2^). L1-SMI cut-offs from our study also showed differences from those based on healthy American populations (men, 34.6cm^2^/m^2^; women, 25.9cm^2^/m^2^)^21^. It might be necessary to explore which L1-SMI cut-off reflects skeletal muscle mass more precisely by comparing the L1-SMI cut-off with grip strength and lean body mass^23^.

Our study demonstrated the accelerated decrease of C-CWMA compared to L1-SMA with aging. One of the notable finding is that C/L1 ratio was not associated with L3-SMI. The accelerated decrease of C-CWMA is rarely reported so far. It could be possible that L3-SMA and L1-SMA were likely to be associated with general medical condition, while C-CWMA would be more likely to be affected by other factors such as morphology of thoracic cage^24^ on top of the general medical condition.

In addition, current smoking seemed to have different effects on decrease of SMA according to the location. Current smoking was associated with accelerated decrease of C-CWMA, while L1-SMI itself was not associated with smoking status. Diaz et al^25^ demonstrated the low muscle mass in chest CT was related to increased mortality in current smokers. Cigarette smoking is a known risk factor for skeletal muscle loss, by inducing proteolysis and inhibiting protein synthesis^26^. However, it is unclear how smoking contributes accelerated decrease of C-CWMA compared to L1-SMA, and causal relationship between smoking and accelerated decrease of C-CWMA could not be determined solely based on our results. Because patients with COPD showed tendency toward having weakened chest wall muscles^27^, one of the possible explanation is that subclinical airflow limitation could also result in decrease of chest wall muscles in current smokers. In this regard, further studies investigating the relationship between smoking, airflow limitation and chest wall muscle should be needed.

In this study, the SMA and SMI of men generally tended to decrease with aging, while this relationship was not confirmed in women. According to results from Magudia et al^19^, age-independence of SMA in women seems not to be obvious in White non-Hispanic and Black. One of the possible explanation for this finding is that age-independence of SMA in women is a result of the difference in lifestyle (such as exercise, diet), which could not be assessed objectively on this retrospective study. Multicenter studies should be needed to investigate whether this finding is Asian characteristic or originates from the difference of lifestyle.

In our study, image reconstruction methods have impact on T12-ESMA and C-CWMA. However, impact of image reconstruction methods was not the main focus of our study, because the magnitude of influence of image reconstruction methods on SMA measurement is not substantial^28^. Recently, deep learning-based body composition segmentation at CT images was validated^29–31^ and this technique could provide robust segmentation from noise^32^. In addition, deep learning also enables researchers to perform time-consuming process more easily^33^. We believe that utilization of deep learning-based technique can bring another breakthrough to body composition measurements.

There were several limitations of this study. First, this was single institutional retrospective study, and only Asian individuals were included in this study. Thus, there could be of unknown bias and the generalization of our results could be limited. The usage of contrast-enhanced CT images might have impact on segmentation of muscle, although small differences of SMI were observed after contrast administration^34^. However, contrast enhancement provided better discrimination of abdominal solid organs, and this was helpful in segmenting skeletal muscles semi-automatically using abdomen CT. Finally, age-dependent changes and the different effect of smoking on each SMA were not fully understood based on this study, because such findings were not correlated with physiologic changes. Further studies to elucidate the relationship of selective muscle atrophy with aging and smoking should be needed in terms of physiologic changes.

In conclusion, L1-SMA could be a reasonable substitute for L3-SMA in chest CT-based study for sarcopenia and suggested cut-offs for sarcopenia using L1-SMI were 39.2cm^2^/m^2^ for men and 27.5cm^2^/m^2^ for women in Asian. Current smoking significantly contributed to accelerated decrease of C-CWMA in general population.

## Methods

This retrospective study was approved by Public Institutional Review Board designated by Ministry of Health and Welfare, Republic of Korea (approval number: P01-202,011–21-015) with a waiver of informed consent, and all experiments were performed in accordance with relevant guidelines and regulations.

### Study population

From January 2017 to December 2019, 918 pairs of contrast-enhanced chest and abdomen LDCT on the same day for healthcare screening were identified in a secondary-referral hospital. In South Korea, there are various types of healthcare screening programs depending on institutes and those who receive these programs are either supported by their companies and local governmental organizations or are examined voluntarily^35,36^. Among them, 29 pairs of examinations were excluded from this study because of following reasons: 1) patients with history of cancer, which could affect the skeletal muscle mass (n = 18), 2) patients with inappropriate arm posture or incomplete scan coverage (i.e. muscles of interest were not fully included)(n = 4), 3) deformity in muscles of interest (i.e. patients who received augmentation mammoplasty)(n = 2), 4) patients who received spine surgery that caused significant beam-hardening artifact at areas of interest (n = 3), and 5) lack of height data (n = 2).

Finally, 780 individuals (mean age, 50.9 ± 10.5 years in men and 51.4 ± 10.8 years in women) with 889 pairs of examinations were included for this study. Among them, 101 individuals received multiple examinations during study period (two CT examinations for 93 individuals and three examinations for 8). Data from multiple examinations were only used to explore annual change rates, and data of the first examination were only used for statistical analysis in individuals with multiple examinations.

### CT protocol, measurement of SMAs and fat area

CT imaging was performed using SOMATOM Definition AS + (Siemens, Erlangen, Germany). Chest and abdomen LDCT were scanned with 50mAs/100kVp and 160mAs/100kVp, respectively, after contrast media administration. To minimize the difference according to contrast-media usage, both contrast-enhanced chest and abdomen LDCT were utilized in this study. For skeletal muscle segmentation, chest CT images with lesser noise were used. During study period, three different image reconstruction methods were applied: 1) 5 mm slice-thickness with I50f. filter (n = 353), 2) 5 mm slice-thickness with I30f. filter (n = 329), and 3) 3 mm slice-thickness with I30f. filter (n = 98). All abdomen CT images were reconstructed with 5 mm slice-thickness with I30f.

Each skeletal muscle was segmented semi-automatically by using open software (3D Slicer Chest Imaging Platform, version 4.10.2), and Hounsfield units of -29 to 150 were considered skeletal muscles^2^. The L3-SMA^2–6^, L1-SMA^11–13^, T12-ESMA^14–16^, C-CWMA^17^ and AA-PMA^11,13,14,18^ were segmented for this study (Supplementary Fig. 2). For segmentation of C-CWMA and AA-PMA, chest CT images were used. In cases that T12 or L1 level was not covered in chest CT, segmentation of T12-ESMA or L1-SMA was done by using abdomen CT images. Because chest CT images with I50f filter showed significant noise at thoracoabdominal junction (i.e. T12 and L1 levels), segmentation of T12-ESMA and L1-SMA were performed with abdomen CT images when available, instead of using chest CT images (Supplementary Fig. 3). The C/L1 ratio was calculated to assess the uneven decrease of skeletal muscles according to their locations. Among three thoracic skeletal muscle areas (AA-PMA, C-CWMA, and T12-ESMA), C-CWMA was selected because of its low annual variability (Fig. 2) and for comprehensive evaluation of thoracic skeletal muscles.

Sarcopenia was defined by using lower 5th percentile values of SMI (SMA/height^2^)^2^ from young individuals (age ≤ 40 years)^21^. To explore the effect of central obesity on skeletal muscle mass, VFA at umbilicus level was also segmented semi-automatically with HU of -250 to -50^37^ using the same software (3D Slicer Chest Imaging Platform), and SFA was also measured if subcutaneous fat was fully covered. If metallic prosthesis was noted at umbilicus level, fat segmentation was not performed because of beam-hardening artifact. TFA was defined as a sum of VFA and SFA. All of segmentation was performed by one author (W.H.L., 6 years of experience in body imaging).

### Collection of clinical information

Anthropometric data (height, body weight, WC), underlying medical diseases (hypertension, diabetes mellitus, dyslipidemia) and smoking status were collected by referring electronic medical records. If exact PY of smoking was available, PY was also recorded.

### Statistical analysis

Normality of the numerical variables was assessed using Kolmogorov–Smirnov test. Numerical variables with normal distribution were provided with mean ± standard deviation, while those not showing normal distribution were provided with median [interquartile range]. To compare multiple SMAs in chest CT with a reference standard (L3-SMA), linear regression was done. Annual change of each skeletal muscle area was calculated with individuals receiving multiple examinations. To explore the effect of scan parameters and reconstruction methods on SMA, multiple regression analysis was performed to adjust the age, sex, BMI, and VFA.

Lower 5th percentile values of SMI from young individuals (age ≤ 40 years)^21^ were selected as Asian specific cut-offs for sarcopenia. Agreements of sarcopenia defined by L3-SMI and chest CT-derived sarcopenia were assessed by Cohen’s Kappa coefficient (κ). The κ was interpreted as follows: (1) slight agreement (κ, 0.01–0.20), (2) fair agreement (κ, 0.21–0.40), (3) moderate agreement (κ, 0.41–0.60), (4) substantial agreement (κ, 0.61–0.80), and (5) almost perfect agreement (κ, 0.81–0.99)^38^. Univariate logistic regression analysis and subsequent multivariate regression analysis with stepwise method were performed to explore factors related to sarcopenia. Factors with *p*-values less than 0.05 on univariate analysis were included on subsequent multivariate regression.

Age-dependent changes of SMAs were evaluated with linear regression and trends were visualized with locally weighted scattered-pot smoother (LOESS) with a span of 0.8^39^. To identify factors related with decrease of C/L1 ratio, linear and subsequent multiple regression analyses with stepwise method were applied.

Statistical analyses were performed using Medcalc (version 15.2, Ostend, Belgium). In this study, *p*-values less than 0.05 were considered statistically significant. Variance inflation factor (VIF) greater than 10 was considered as presence of multicolinearity, and variables with VIF greater than 10 were not included for the multivariate analysis.

## Supplementary Information

Supplementary Figures.

## Data Availability

Data of our study population could be accessed as a separate excel file.
