# Supplementary material for: Validation for measurements of skeletal muscle areas using low-dose chest computed tomography
Source: Sci Rep. 2022 Jan 10;12:463. doi: 10.1038/s41598-021-04492-1 (PMC8748601; doi:10.1038/s41598-021-04492-1)
Supplement: Supplementary file 1 — Supplementary Figures. [file 41598_2021_4492_MOESM1_ESM.pdf]

Validation for measurements of skeletal muscle areas using low-dose chest computed tomography (LDCT)

Woo Hyeon Lim [1,2], Chang Min Park [2,3,4,5*]

1 Department of Radiology, Namwon Medical Center, Namwon-si, Jeollabuk-do, Korea;

2 Department of Radiology, Seoul National University College of Medicine, Seoul, Korea;

3 Institute of Radiation Medicine, Seoul National University Medical Research Center, Seoul, Korea;

4 Cancer Research Institute, Seoul National University College of Medicine, Seoul, Korea;

5 Institute of Medical and Biological Engineering, Medical Research Center, Seoul National University, Seoul, Korea

* Corresponding author: Chang Min Park, M.D.

Department of Radiology, Seoul National University College of Medicine, Seoul, Korea;

101 Daehak-ro, Chongno-gu, Seoul, 03080, Korea.

Tel: 82-2-2072-0367, Fax: 82-2-743-7418

E-mail: cmpark.morphius@gmail.com

Type of manuscript: original research

Supplementary Materials

Supplementary Figure 1. Age-dependent changes of skeletal muscle areas (SMAs) and indices (SMIs): a) L3-SMA (men, $p<0.001$; women, $p=0.002$), L3-SMI (men, $p=0.001$; women, $p=0.080$); b) L1-SMA (men, $p<0.001$; women, $p=0.417$), L1-SMI (men, $p=0.495$; women, $p<0.001$); c) T12-ESMA (men, $p<0.001$; women, $p=0.874$), T12-ESMI (men, $p=0.007$; women, $p<0.001$); d) C-CWMA (men, $p<0.001$; women, $p<0.001$), C-CWMI (men, $p<0.001$; women, $p=0.774$); e) AA-PMA (men, $p<0.001$; women, $p=0.680$), AA-PMI (men, $p=0.002$; women, $p<0.001$). In men, all SMAs and SMIs except L1-SMI decreased with aging ($p<0.05$), even after adjusting body-mass index (BMI). In women, L3-SMA and C-CWMA decreased with aging ($p<0.05$), while L1-SMI, T12-ESMI and AA-PMI increased with aging ($p<0.05$). However, all SMIs were irrelevant to aging in women after adjusting BMI ($p>0.05$).

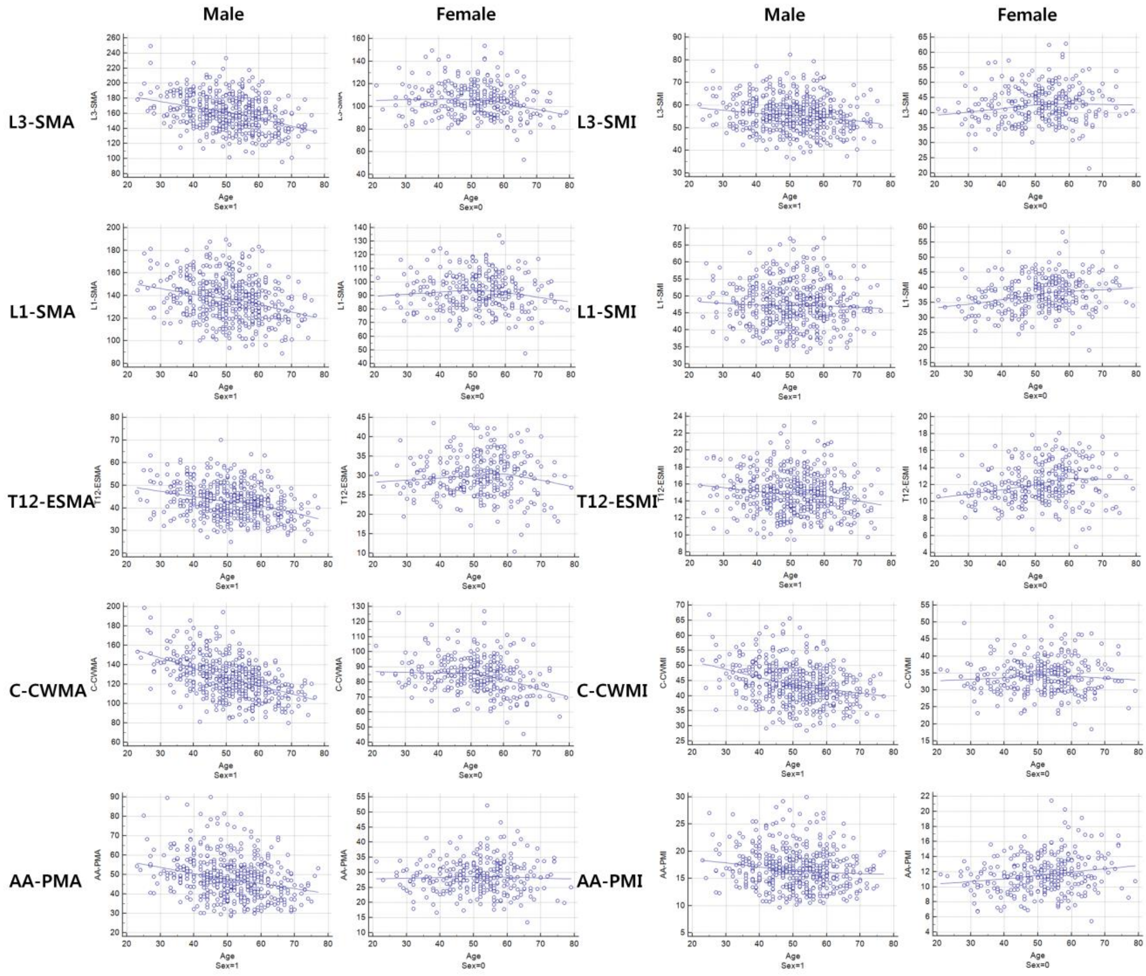

Note> L3-SMA/SMI=SMA/SMI at 3rd lumbar vertebra level, L1-SMA/SMI=SMA/SMI at 1st lumbar vertebra level, T12-ESMA/ESMI= erector spinae muscle area/index at 12th thoracic vertebra level, C-CWMA/CWMI=chest wall muscle area/index at carina level, AA-PMA/PMI=pectoralis muscle area/index just above aortic arch level.

Supplementary Figure 2. Examples of region of interest selection using chest CT: a) skeletal muscle area at L1 level (L1-SMA; usually contains dorsal back muscle groups, diaphragmatic crus, intercostal muscles and abdominal wall muscles), b) erector spinae muscle area at T12 level (T12-ESMA), c) chest wall muscle area at carina level (C-CWMA; usually contains dorsal back muscle groups, subscapularis muscles, serratus anterior muscles, pectoralis muscles, and intercostal muscles), d) pectoralis muscle area just above aortic arch level (AA-PMA), e) skeletal muscle area at L3 level (L3-SMA), and f) visceral fat and subcutaneous fat areas at umbilicus level.

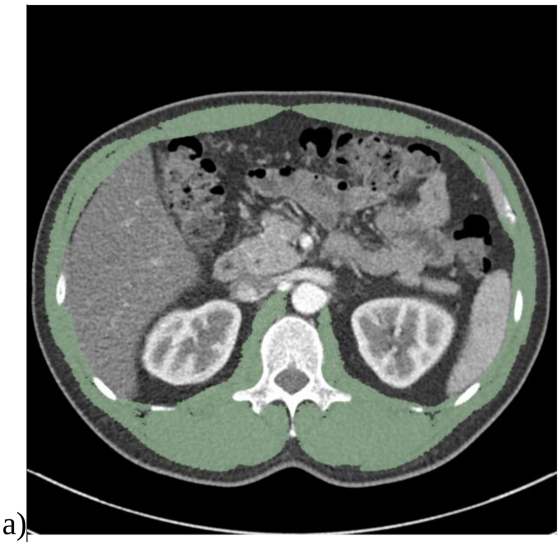
a)

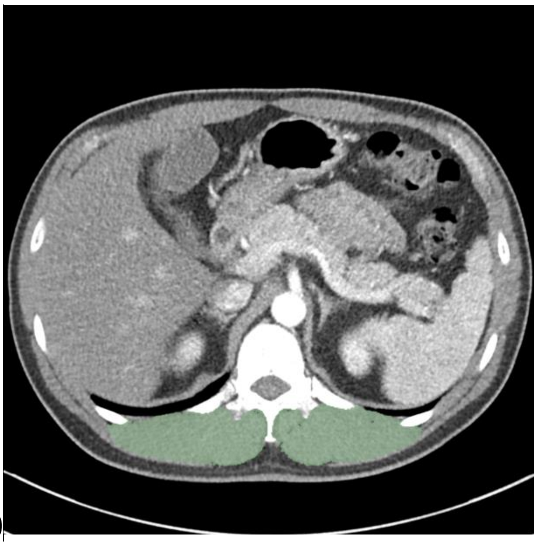
b)

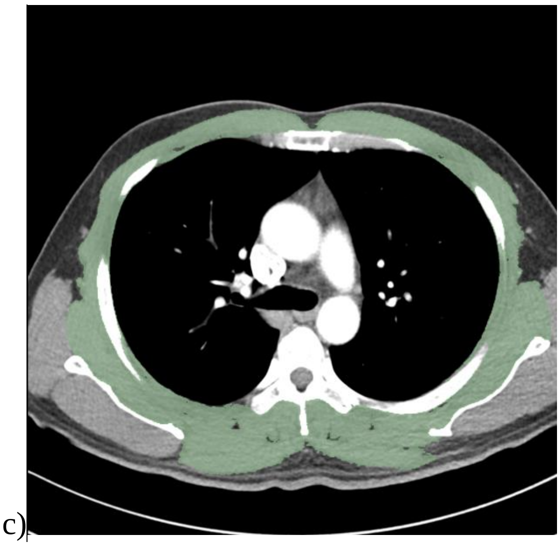
c)

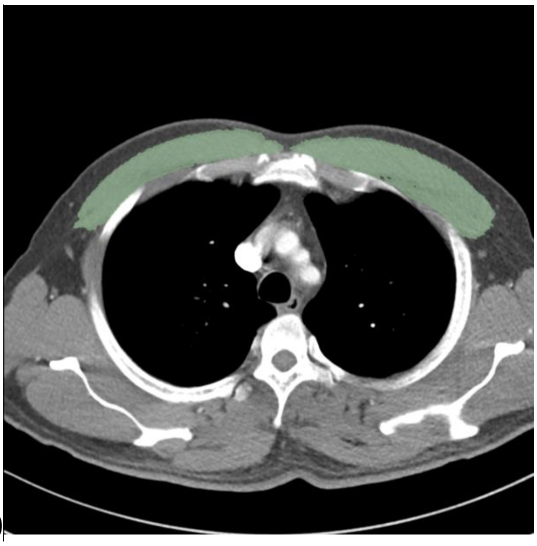
d)

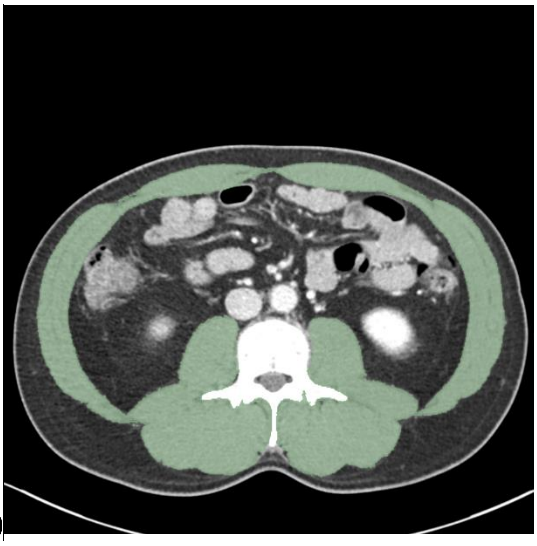

e)

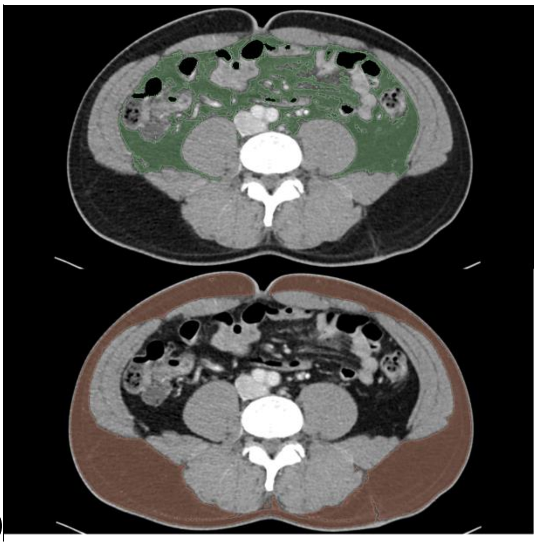

f)

Supplementary Figure 3. Examples of chest CT images according to image reconstruction methods: a) L1-SMA with 5mm slice-thickness with I50f filter, b) L1-SMA with 5mm slice-thickness with I30f filter, and c) L1-SMA with 3mm slice-thickness with I30f filter.

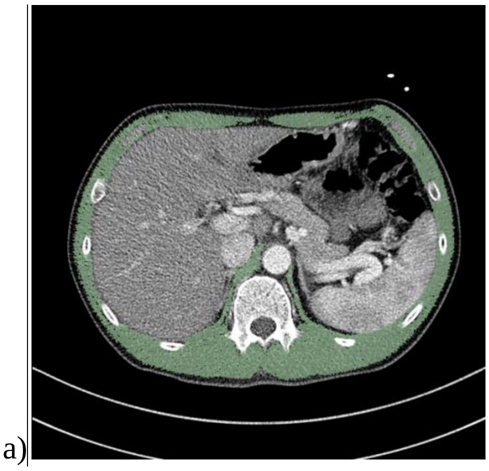

a)

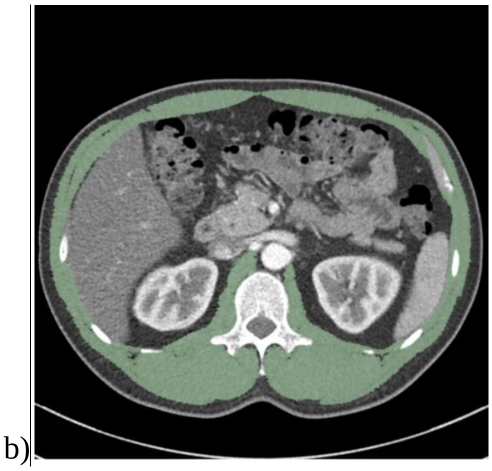

b)

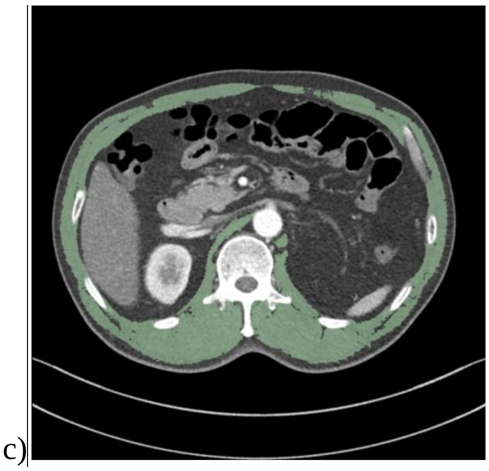

c)
